# Low-Dose Naltrexone in Chronic Pain Management: Mechanisms, Evidence, and Clinical Implications

**DOI:** 10.3390/jpm16030151

**Published:** 2026-03-06

**Authors:** Alyssa McKenzie, Tiffany Bittar, Rachel Dombrower, Dupinder Raman, Hatim Hussain, Nitchanan Theeraphapphong, Sophia M. McKenzie, Alaa Abd-Elsayed

**Affiliations:** 1 School of Medicine, St. George’s University, West Indies 11739, Grenadahhussai4@sgu.edu (H.H.);; 2 School of Medicine, Uniformed Services University of the Health Sciences, Bethesda, MD 20814, USA; 3 Department of Anesthesiology, University of Wisconsin School of Medicine and Public Health, Madison, WI 53792, USA

**Keywords:** low-dose naltrexone, chronic pain, central sensitization, neuroinflammation, microglia, Toll-like receptor 4, nociplastic pain, multimodal pain management

## Abstract

Chronic pain imposes a substantial burden on global health and remains challenging to manage, despite ongoing advances in pharmacologic and interventional therapies. Recognition of chronic pain as a condition driven by central sensitization and neuroimmune dysregulation has prompted interest in therapies that target these mechanisms rather than peripheral nociception alone. Low-dose naltrexone (LDN), administered at doses substantially lower than those used for opioid or alcohol use disorders, has emerged as a repurposed treatment with potential analgesic and anti-inflammatory properties. This review summarizes the pharmacologic characteristics of LDN, with emphasis on its proposed mechanisms involving transient opioid receptor blockade, modulation of microglial activation, Toll-like receptor signaling, and central neuroimmune pathways. Available clinical evidence evaluating LDN across a range of chronic pain conditions, such as fibromyalgia, neuropathic pain syndromes, inflammatory and autoimmune disorders, headache disorders, and other centralized pain states, is critically reviewed. Although early trials, observational studies, and case series suggest potential benefit in selected populations, the overall evidence base remains limited, heterogeneous, and characterized by variability in dosing strategies and outcome measures. Safety, tolerability, and practical considerations relevant to contemporary pain practice are discussed, including interactions with opioid therapy and challenges related to off-label use. Finally, key gaps in the current evidence and priorities for future research are highlighted, underscoring the need for larger, well-designed randomized trials and mechanism-informed studies to better define LDN’s role in multimodal chronic pain management.

## 1. Introduction

Chronic pain is associated with functional impairment, reduced quality of life, and substantial healthcare utilization [1,2]. Despite advances in pharmacologic and interventional management, many patients experience inadequate symptom control or adverse effects that limit long-term therapy [3,4]. Concerns regarding the limited utility and risk profile of long-term opioid therapy have prompted interest in multimodal and mechanism-oriented approaches [2,4].

Interest has grown in repurposing medications with established safety profiles for analgesic indications. Naltrexone, a competitive opioid receptor antagonist traditionally used for opioid and alcohol use disorders, has emerged as a candidate therapy when administered at substantially lower doses [5]. Low-dose naltrexone (LDN), typically prescribed at 0.5–4.5 mg daily, is used off-label in chronic pain and inflammatory conditions [6,7].

Proposed effects of LDN extend beyond opioid receptor antagonism and include modulation of neuroimmune signaling, microglial activity, and central sensitization pathways [8,9]. These mechanisms align with contemporary models of chronic pain involving altered central nociceptive processing.

Chronic pain is not a single entity but encompasses distinct pathophysiological phenotypes, including nociceptive, neuropathic, and nociplastic pain [10]. Neuropathic and nociplastic pain are characterized by altered central nociceptive processing, central sensitization, and neuroimmune activation rather than ongoing peripheral tissue injury alone [9,11]. In these phenotypes, traditional agents such as nonsteroidal anti-inflammatory drugs or opioids often demonstrate limited and inconsistent efficacy [2,3]. Because the proposed rationale for low-dose naltrexone centers on modulation of microglial activity, Toll-like receptor signaling, and central sensitization pathways, its theoretical positioning aligns more closely with neuropathic and nociplastic pain mechanisms than with purely peripheral inflammatory nociceptive pain [8,9,12].

Clinical interest in LDN has been driven by early studies and case series suggesting benefit across diverse chronic pain syndromes, including fibromyalgia, neuropathic pain, inflammatory conditions, and headache disorders [6,12]. At the same time, there is a lack of standardized guidance regarding patient selection, dosing strategies, and clinical integration, as the evidence base remains limited and heterogeneous [12].

This narrative review examines the use of low-dose naltrexone across major chronic pain phenotypes, including fibromyalgia, neuropathic pain syndromes, complex regional pain syndrome, musculoskeletal pain, headache disorders, chronic pelvic pain, inflammatory and autoimmune-related pain, post-infectious pain, and cancer-related pain. The review addresses three core clinical questions: what biological pathways have been proposed and what level of evidence supports them; what clinical efficacy data exist across pain conditions; and how LDN may be integrated into practice with respect to dosing, opioid interactions, and perioperative management. This review does not provide formal systematic evidence grading or meta-analysis and does not address ultra-low-dose naltrexone regimens or non-pain indications outside the scope of chronic pain management.

## 2. Methods

This manuscript was conducted as a narrative review synthesizing current mechanistic and clinical evidence regarding low-dose naltrexone (LDN) in chronic pain management. A structured literature search was performed in PubMed/MEDLINE and ClinicalTrials.gov through January 2026 using combinations of the terms “low-dose naltrexone,” “LDN,” and chronic pain-related conditions, including fibromyalgia, neuropathic pain, complex regional pain syndrome, migraine, pelvic pain, and inflammatory disorders. Reference lists of relevant review articles and primary studies were manually screened to identify additional publications. Studies and registered trials were selected based on relevance to LDN use in chronic pain and proposed neuroimmune pathways. Given the narrative design, conditions were selected based on frequency of reporting in the LDN pain literature and clinical relevance to chronic non-cancer pain syndromes encountered in pain medicine practice. Non-pain indications and dermatologic or mucosal autoimmune conditions were not comprehensively reviewed, as they fall outside the scope of chronic pain management. This review focuses specifically on chronic non-cancer pain conditions.

## 3. Pharmacology and Dosing

### 3.1. Standard Indications and Dose Distinctions

Naltrexone is a competitive opioid receptor antagonist with high affinity for μ-opioid receptors and lower affinity for κ- and δ-opioid receptors. At standard dosing (50 mg taken orally once daily or 380 mg administered intramuscularly once monthly), naltrexone is used for the treatment of opioid and alcohol use disorders [13]. It functions as a long-acting opioid receptor antagonist, providing sustained suppression of opioid receptor signaling.

LDN is prescribed off-label at doses ranging between 0.5 and 4.5 mg daily [14]. At these dosage levels, LDN is thought to produce transient opioid receptor blockade, which has been hypothesized to be followed by changes in endogenous opioid signaling and modulation of central neuroimmune pathways [14]. In contrast to sustained antagonism with standard dosing, low-dose regimens are theorized to produce short-lived receptor occupancy; however, sustained endogenous opioid upregulation has not been demonstrated in human clinical studies [8,14,15]. Key pharmacologic and dosing differences between standard-dose naltrexone and low-dose naltrexone are summarized in Table 1.

### 3.2. Pharmacokinetics at Low Doses

Following oral administration, naltrexone undergoes extensive first-pass hepatic metabolism to 6-β-naltrexol, a longer-acting active metabolite with reduced central opioid receptor effects relative to naltrexone [16]. The elimination half-life of naltrexone is approximately 4 h, whereas 6-β-naltrexol has a longer half-life of approximately 13 h [16]. At low doses, systemic exposure is reduced, resulting in transient opioid receptor occupancy rather than sustained antagonism.

At LDN doses, plasma concentrations are significantly lower than those achieved with standard dosing, resulting in short-lived opioid receptor occupancy [16]. This transient antagonism is hypothesized to allow for downstream compensatory upregulation of endogenous opioid signaling and modulation of glial cell activity after receptor blockade resolves, a process thought to be central to its proposed analgesic and anti-inflammatory effects [8,16]. However, the relationship between plasma concentration, receptor occupancy, and clinical effect at low doses has not been definitively established.

### 3.3. Dose–Response Considerations

Available evidence suggests that LDN may exhibit a nonlinear or biphasic dose–response relationship, with therapeutic effects occurring within a relatively narrow dosing window [14]. Doses that are too low may be subtherapeutic [17,18]. In contrast, higher doses approaching standard naltrexone dosing ranges may negate the therapeutic effects by producing sustained opioid antagonism and interfering with endogenous analgesic pathways [5,16]. Most studies report benefits at doses between 3.0 and 4.5 mg daily, although some patients appear to respond to lower doses, particularly those with heightened sensitivity to neuropsychiatric side effects [17].

### 3.4. Dosing Strategies in Clinical Practice

Low-dose naltrexone is typically initiated at 0.5–1.5 mg once daily and titrated toward a target range of 3–4.5 mg daily [1,19]. Dose escalation is commonly performed in increments of 0.5–1.5 mg at intervals of approximately 1–2 weeks. A frequently used schedule increases from 1.5 mg daily to 3 mg daily after 1–2 weeks, followed by 4.5 mg daily after an additional 1–2 weeks if tolerated. Some clinicians initiate at 0.5^−1^ mg daily in patients with heightened sensitivity to sleep disturbance or neuropsychiatric symptoms and use smaller incremental increases. Published clinical trials have not employed a uniform titration protocol, and no randomized study has directly compared specific titration strategies or step durations. Accordingly, titration is individualized based on tolerability, adverse effects, and clinical response.

Commercially available naltrexone tablets are formulated at higher doses; therefore, LDN is obtained through compounding pharmacies to allow precise dosing and gradual titration [20]. Clinical response is generally assessed after several weeks of stable dosing, as LDN does not produce immediate analgesic effects [5,7].

### 3.5. Interaction with Opioid Therapy

A key factor to consider in LDN therapy is its interaction with opioid agonists. Even at low doses, naltrexone antagonizes opioid receptors and can reduce opioid analgesic efficacy [21]. It may also precipitate withdrawal symptoms in opioid-dependent patients [13,21]. For the purposes of this review, “chronic opioid therapy” refers to the regular daily use of opioid agonists for ≥3 months for persistent pain, consistent with common pain medicine definitions. Patients receiving stable daily opioid therapy should be considered at risk for reduced analgesic efficacy or withdrawal if LDN is initiated without prior tapering.

Accordingly, LDN should not be initiated in patients actively receiving chronic daily opioid therapy [13,21]. Most published protocols recommend complete opioid discontinuation prior to LDN initiation, commonly for a minimum of 7–10 days, although high-quality comparative data defining the optimal washout interval are limited [4]. In clinical practice, decisions regarding opioid tapering should be individualized based on baseline dose, duration of therapy, and risk of withdrawal.

Most studies evaluating LDN exclude patients receiving opioids [5,19]. This limitation significantly impacts the generalizability of LDN to real-world pain populations, where opioid use remains prevalent. Ultra-low-dose naltrexone (ULDN) refers to microgram-range dosing, typically between approximately 0.1 and 100 micrograms per day. Unlike LDN, ULDN has primarily been investigated as an adjunct to ongoing opioid therapy to attenuate opioid tolerance, opioid-induced hyperalgesia, or withdrawal symptoms [22]. Proposed mechanisms in this context involve modulation of opioid receptor signaling [22,23]. In contrast, milligram-range LDN (0.5–4.5 mg daily) is administered as a standalone therapy and has been proposed to exert effects through transient opioid receptor blockade and modulation of central neuroimmune pathways, including microglial signaling [8,24]. These regimens differ in dose range, clinical context, and proposed mechanisms, and findings from ULDN studies should not be extrapolated to milligram-range LDN in chronic pain management.

## 4. Mechanisms Underlying Analgesic Effects

### 4.1. Transient Opioid Receptor Blockade and Endogenous Opioid Signaling

Low-dose naltrexone has been proposed to exert analgesic effects through transient opioid receptor blockade [23,25]. This proposed model suggests that brief receptor antagonism may be followed by changes in endogenous opioid activity, including β-endorphin and enkephalin signaling [1,8,15]. In contrast to standard-dose naltrexone, which produces sustained receptor antagonism, low-dose regimens are theorized to result in short-lived receptor occupancy rather than continuous blockade [8,15,25].

This hypothesis is based largely on pharmacologic theory and limited preclinical evidence. Sustained upregulation of endogenous opioids or receptor sensitization has not been directly demonstrated in human chronic pain populations treated with LDN [5,12]. The endogenous opioid rebound model should therefore be considered a proposed explanation rather than a clinically confirmed mechanism [16].

### 4.2. Microglial Modulation and Neuroimmune Effects

LDN has also been proposed to modulate neuroimmune signaling through effects on microglial activation [8,9]. Activated microglia release pro-inflammatory cytokines, chemokines, and excitatory mediators that enhance nociceptive transmission within the central nervous system [26,27]. In preclinical models, naltrexone and related compounds reduce microglial activation and decrease the production of inflammatory mediators [28].

However, evidence supporting microglial modulation in human chronic pain populations treated with LDN remains indirect. Clinical trials reporting symptom improvement in conditions such as fibromyalgia or complex regional pain syndrome have not included biomarker, cerebrospinal fluid, or neuroimaging confirmation of reduced microglial activation [8]. Therefore, microglial modulation should be regarded as a preclinical mechanism with hypothesized relevance to human disease rather than a directly verified clinical pathway.

### 4.3. TLR-4 Antagonism

Beyond opioid receptor interactions, naltrexone and its stereoisomers antagonize Toll-like receptor 4 (TLR4) signaling in in vitro and animal models [29,30]. TLR4 activation promotes microglial-driven inflammatory cascades that contribute to pain hypersensitivity in experimental systems [24,31]. Preclinical studies show that TLR4 blockade reduces inflammatory mediator release and mechanical allodynia in animal models [24,29].

The contribution of TLR4 antagonism to analgesia in human chronic pain has not been established. No clinical trials have directly demonstrated modulation of TLR4 signaling in patients receiving LDN. TLR4 antagonism should therefore be interpreted as a preclinical finding with translational potential rather than a clinically established mechanism.

### 4.4. Effects on Central Sensitization

Central sensitization refers to increased excitability of central nervous system neurons, leading to amplified pain signaling and impaired inhibitory modulation [10,11]. This process is implicated in conditions such as fibromyalgia and chronic pelvic pain [32].

LDN has been proposed to influence central sensitization through combined effects on opioid signaling and neuroimmune pathways, based on preclinical observations [24,33]. Clinical studies report improvements in pain severity and related symptoms in selected chronic pain populations treated with LDN [7,8,22]. However, these studies have not directly measured central sensitization using quantitative sensory testing, neuroimaging, or validated biomarkers. Improvements in symptoms should therefore not be interpreted as direct evidence of reversal of central sensitization. The relationship between LDN and central sensitization in humans remains inferential.

### 4.5. Summary of Pathophysiologic Evidence Levels

**Figure 1:**
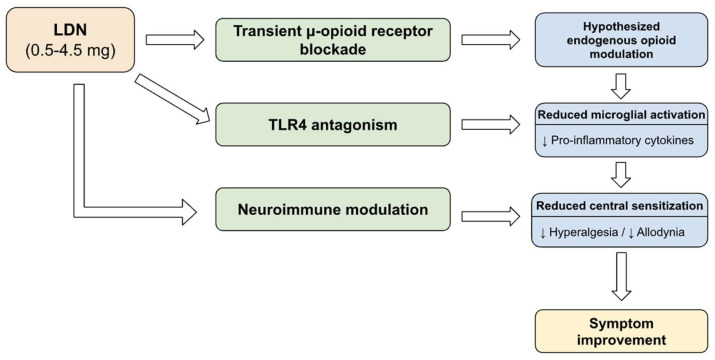
Proposed mechanisms of action of low-dose naltrexone in chronic pain.

Among the proposed biological pathways, TLR4 antagonism and microglial modulation are supported primarily by in vitro and animal data [24,29,30,31]. Human studies have not confirmed these mechanisms using biomarkers or neuroimaging. The endogenous opioid rebound model is derived from pharmacologic theory and limited preclinical findings [16,25], and sustained increases in endogenous opioid activity have not been demonstrated in clinical trials. Although randomized and observational studies report symptom improvement in selected pain conditions [7,8,22], none have directly measured the proposed molecular or cellular pathways. Translation of these findings to human chronic pain remains inferential. A schematic overview of the proposed biological pathways of low-dose naltrexone is presented in Figure 1.

## 5. Clinical Evidence in Chronic Pain Conditions

LDN has been evaluated across multiple chronic non-cancer pain phenotypes. Table 2 summarizes representative randomized and observational primary studies, as well as key secondary syntheses (systematic reviews and meta-analyses), evaluating LDN in chronic non-cancer pain conditions. It is not intended to be an exhaustive registry of all published reports. For secondary syntheses, the number and type of included primary studies are specified to clarify the breadth of underlying evidence.

### 5.1. Fibromyalgia

Research has explored the potential of low-dose naltrexone in managing fibromyalgia, a nociplastic pain condition characterized by central sensitization and widespread pain [46,47]. Preliminary clinical studies, including small RCTs, suggest that LDN (typically 3–4.5 mg daily) may reduce pain intensity and other fibromyalgia symptoms compared with placebo in some patients, although sample sizes have been limited and effect sizes modest [34,36]. Importantly, the largest parallel-group randomized trial to date [19] did not demonstrate statistically significant superiority over placebo for its primary endpoint, underscoring the variability and modest magnitude of effect observed across studies. Consistent with these findings, a recent meta-analysis of randomized controlled trials evaluating low-dose naltrexone across chronic pain syndromes found no significant overall analgesic benefit but did identify modest pain reduction in fibromyalgia-specific analyses, highlighting both condition-specific effects and the limitations of the current evidence base [37]. Retrospective and uncontrolled data suggest possible benefit in subsets of patients; however, these findings are limited by small sample sizes, short follow-up duration, and the risk of selection bias [6,36]. Observed symptom improvements have been hypothesized to relate to neuroimmune modulation; however, these pathways have not been directly confirmed in fibromyalgia trials [8,10,28].

### 5.2. Neuropathic Pain

LDN has been investigated in several conditions involving neuropathic pain, although well-designed randomized clinical trials remain limited [12,48]. In a randomized, double-blind, active-controlled crossover trial of LDN (1–4 mg/day) compared with amitriptyline in painful diabetic peripheral neuropathy (DPN), pain reduction was comparable between groups; however, the study was limited by small sample size and short duration, and superiority over established therapies was not demonstrated [38].

There are no randomized controlled trials specifically evaluating LDN for small fiber neuropathy (SFN) [12,48,49]. Available data derive from observational reports and chart-based analyses in broader chronic pain cohorts in which SFN is present as a comorbidity [39,50,51]. These uncontrolled findings suggest that some patients with SFN receiving LDN for overlapping chronic pain conditions may report symptomatic improvement; however, efficacy cannot be established. The use of LDN in SFN should therefore be considered extrapolated from broader neuropathic and centralized pain literature rather than supported by condition-specific clinical trials. In related peripheral neuropathic pain conditions, small observational studies report reductions in pain and sensory symptoms, including in neuropathic corneal pain, although these data are also uncontrolled [52].

There are currently no randomized controlled trials of LDN for postherpetic neuralgia (PHN) [7,12]. The proposed benefit is based on preclinical evidence of Toll-like receptor 4 (TLR4) antagonism and modulation of glial activation [9,31], but this has not been confirmed in PHN-specific clinical studies [7,12].

Similarly, data on LDN for central neuropathic pain syndromes such as post-stroke pain and spinal cord injury are primarily preclinical, with limited clinical evidence available [12,53]. Although animal models suggest effects on microglial activation and central inflammatory signaling [24,54], clinical trials in these populations have not been published [12].

In trigeminal neuropathic pain, retrospective case series report reductions in subjective pain intensity in patients with treatment-resistant symptoms [39]. However, these findings are derived from uncontrolled designs and cannot establish efficacy [39,55].

### 5.3. Complex Regional Pain Syndrome

Complex regional pain syndrome (CRPS) is a neuropathic and central sensitization pain condition in which LDN has been used off-label [33,40,56]. Available evidence consists primarily of small retrospective cohorts and case series. In these reports, pain outcomes were typically assessed using numerical rating scales (NRS) or visual analog scales (VAS), with some patients reporting clinically meaningful reductions in pain intensity following LDN initiation [40,57]. However, sample sizes have been small, response rates have varied, and standardized responder definitions were not consistently applied. No placebo-controlled randomized trials have evaluated LDN specifically in CRPS, limiting conclusions regarding efficacy and magnitude of benefit [9,12,58]. While proposed mechanisms involve modulation of glial-mediated neuroinflammation and central sensitization, clinical confirmation in CRPS remains lacking [9,12,58].

### 5.4. Musculoskeletal Pain Syndromes

The data supporting LDN for musculoskeletal pain conditions, such as osteoarthritis and low back pain, are limited and largely preliminary [41,59]. A randomized, double-blind crossover trial in arthritis did not demonstrate consistent improvement compared with placebo, highlighting limited evidence of benefit in predominantly nociceptive pain conditions [41]. Observational data suggest possible symptom improvement in some patients; however, findings have been inconsistent and effect sizes variable [41,59]. An observational dose-optimization study in patients with chronic musculoskeletal pain further demonstrated substantial interindividual variability in response to LDN, with symptom improvement reported in a subset of patients but no consistent effect across the cohort [60].

### 5.5. Headache and Migraine Disorders

Low-dose naltrexone has been investigated for the treatment of headache and migraine conditions, especially in cases that are resistant to standard therapies [61,62]. A case report noted decreased headache frequency and intensity in a patient with chronic migraine and multiple sclerosis who received 4.5 mg/day of LDN [61]. Mechanistic theories propose that the advantages of LDN may be linked to Toll-like receptor-4 antagonism and microglial inhibition, both of which are significant to migraine pathology [62]. In the absence of randomized controlled trials, current evidence is limited to isolated case reports and small series, precluding conclusions regarding efficacy.

### 5.6. Chronic Pelvic Pain Disorders

Within chronic pelvic pain conditions, emerging evidence has discussed LDN as a therapy for endometriosis-associated pain, interstitial cystitis, and vulvodynia [12,42,63,64]. In addition to published observational studies, small clinical case series in patients with refractory vulvodynia, as well as recent conference-reported pilot data, have described patient-reported reductions; however, these findings are based on small, uncontrolled studies and conference abstracts without long-term follow-up [42,62,63,64]. These reported improvements extend beyond isolated pelvic pain syndromes and overlap with broader central and nociplastic pain conditions [12,62]. The proposed mechanisms underlying LDN’s effects in chronic pelvic pain center on immune modulation, microglial inhibition, and altered central pain processing [12,42].

### 5.7. Inflammatory and Autoimmune-Related Pain

Low-dose naltrexone has been investigated in inflammatory and immune-mediated conditions, most notably inflammatory bowel disease, multiple sclerosis, and rheumatoid arthritis, with additional interest in other chronic inflammatory disorders [6,43,65,66]. For instance, analysis of the current literature has shown that adding LDN to patient-specific disease regimens reduces the daily use of NSAIDs and opioid use [43]. Some studies report improvement in disease-related symptoms; however, the evidence is derived largely from small trials or registry analyses and remains heterogeneous [14,43]. The mechanism of LDN effects in inflammatory and autoimmune pain is generally attributed to neuroimmunomodulatory and anti-inflammatory processes, particularly through glial and cytokine modulation, rather than classic opioid-mediated analgesic pathways [7,8].

### 5.8. Post-Infectious Pain

Low-dose naltrexone has been evaluated in post-infectious and post-inflammatory pain states, including pain following viral illness and inflammatory injury [44,45]. Reported clinical contexts include post-COVID-19 condition and myalgic encephalomyelitis, which are characterized by persistent pain, fatigue, and immune dysregulation [44,45]. LDN has been proposed to modulate maladaptive neuroimmune signaling in these conditions [43,44]. Small observational cohorts report reductions in pain and fatigue, with some patients describing improvement in functional measures [44,45]. Available data are limited to small, uncontrolled observational cohorts, and comparative efficacy relative to established treatments has not been determined [44,45].

### 5.9. Clinical Trial Evidence and Ongoing Investigations

Clinical trial activity evaluating LDN in chronic pain and related conditions has included studies across fibromyalgia, chronic pelvic pain syndromes, post-infectious fatigue states, central sensitivity syndromes, and HIV-associated pain. Randomized trials in fibromyalgia (NCT03970330, NCT04313972, and NCT04270877) have primarily evaluated changes in pain intensity and Fibromyalgia Impact Questionnaire (FIQ) scores as primary endpoints, while an ongoing phase IV study (NCT04739995) includes pain severity and inflammatory biomarkers. Trials in bladder pain syndrome (NCT04450316) have assessed pelvic pain and urinary symptom scores, and studies in post-viral fatigue and post-COVID conditions (NCT05430152) have focused on fatigue severity, pain intensity, and global symptom burden. An ongoing HIV-associated pain study (NCT05537935) evaluates neuropathic pain intensity and quality-of-life measures.

## 6. Safety and Tolerability

### 6.1. Common Adverse Effects

Common adverse effects of LDN are generally mild and include headaches, sleep disturbances, nausea, vomiting, decreased appetite, joint pain, muscle cramps, and dizziness [6,67,68,69]. LDN has been associated with a low incidence of severe adverse effects, but data are limited [19].

### 6.2. Neuropsychiatric Effects

Neuropsychiatric effects are among the most common, mild, and reversible adverse side effects associated with low-dose naltrexone. Such side effects include a rapid onset of vivid dreams, insomnia, headaches, anxiety, and mood changes [8]. It is important to note, however, that anxiety associated with LDN may arise from temporary opioid receptor blockade, which reduces endogenous opioid signaling and can produce symptoms resembling mild opioid withdrawal [8]. Paradoxically, some studies have noted an improved mood and sleep quality in patients taking LDN [35]. Severe psychiatric adverse events have not been commonly documented in studies of LDN, with most reported neuropsychiatric adverse side effects described as mild and temporary [8].

### 6.3. Hepatic Considerations

Naltrexone has often been considered hepatotoxic; however, this reputation primarily reflects dose-dependent elevations in hepatic transaminases rather than definitively established clinically apparent liver failure [70,71]. The reported hepatic transaminase elevations associated with standard-dose naltrexone therapy are typically mild and self-limiting [71]. In contrast, clinically significant hepatic enzyme elevations have rarely been reported with LDN, and available data suggest that hepatotoxicity is uncommon at low doses [6,8]. As such, routine liver function monitoring may not be necessary in patients without underlying liver disease [6,8]. Nonetheless, baseline liver assessment may be considered in patients with known hepatic dysfunction, alcohol use disorder, or concurrent use of hepatotoxic medications [72].

### 6.4. Overall Safety Profile

Low-dose naltrexone overall has demonstrated a favorable safety profile in clinical studies [50]. Although much of the safety data for naltrexone is derived from standard or high-dose use, available evidence suggests that adverse effects associated with low-dose naltrexone are generally mild and self-limiting [6,8]. However, long-term safety data remain limited [6,8]. The most commonly reported adverse side effects have been of neuropsychiatric origin, including sleep disturbances, anxiety, vivid dreams, and insomnia [6,8]. More serious adverse effects, such as clinically significant hepatic toxicity, have not commonly been reported, and naltrexone has not been definitively linked to cases of clinically apparent liver injury [70]. Given its benign side effect profile and minimal potential for abuse, LDN may represent a safe option within the approach to chronic pain management [8,73].

### 6.5. Renal Function and Special Populations

Naltrexone undergoes hepatic metabolism with renal excretion of its metabolites [16]. Specific dose adjustments for low-dose naltrexone (LDN) in chronic kidney disease have not been established, and clinical pharmacokinetic data in advanced renal impairment are limited. Caution may be warranted in patients with moderate to severe renal dysfunction. Data regarding LDN use in older adults, pregnancy, and lactation remain limited [13,73]. Age-related changes in hepatic and renal function may justify cautious titration in older patients. Safety data for LDN in pregnancy and breastfeeding are insufficient, and use in these populations should be individualized.

Beyond opioid agonists, naltrexone is not a major cytochrome P450 substrate, inducer, or inhibitor [13,16], and clinically significant pharmacokinetic interactions are not commonly reported. However, monitoring for additive central nervous system effects may be appropriate when combined with other centrally acting medications.

## 7. Practical Considerations for Pain Practice

### 7.1. Patient Selection

Given its indirect pain-modulating effects rather than direct analgesia, low-dose naltrexone may be most appropriate for patients with chronic pain syndromes characterized by central sensitization or neuroinflammatory mechanisms, particularly those who have experienced limited relief from conventional analgesics [8,12]. Conditions such as fibromyalgia, Crohn’s disease, multiple sclerosis, and complex regional pain syndrome have been the most frequently studied [6,8,35,74]. Because proposed pathways include modulation of central sensitization and neuroimmune signaling, LDN may be considered in patients with multiple coexisting chronic pain conditions, particularly when these conditions share features of centralized or inflammatory pain processing. In such cases, a single adjunctive therapy may address overlapping symptom domains rather than targeting each condition separately. Ideally, LDN should be avoided in patients who are actively receiving opioid agonist therapy, as opioid receptor antagonism may cause withdrawal symptoms or reduce opioid analgesic efficacy [6,13]. Caution may be taken in patients with a history of psychiatric comorbidities, as neuropsychiatric effects, although transient, have been reported and noted to have a rapid onset in the initial phases of treatment [8].

### 7.2. Initiation and Titration

LDN is typically initiated at 0.5–1.5 mg daily and titrated gradually to 3–4.5 mg daily. Dose increases are commonly performed every 1–2 weeks, although schedules vary across studies and clinical practice. No consensus titration protocol has been established in the literature, and no trial has directly compared fixed-dose versus titrated initiation strategies. Titration is therefore individualized based on tolerability and observed symptom response.

### 7.3. Timing of Administration

LDN is most commonly administered once daily, with nighttime dosing historically recommended [8]. This practice was based on the theoretical premise that transient opioid receptor blockade might be followed by endogenous opioid rebound during nocturnal cycles [8,57]. However, this mechanism has not been confirmed in human studies, and no clear evidence demonstrates superior analgesic efficacy with nighttime compared to daytime administration [75]. Accordingly, dosing time should be individualized based on patient tolerability, particularly in relation to sleep-related adverse effects such as insomnia or vivid dreams [8].

### 7.4. Monitoring and Assessment of Response

Clinical response to LDN is primarily assessed using patient-reported outcomes, including changes in pain levels, fatigue, sleep quality, and overall symptom burden [8,35]. Since LDN does not act as a typical analgesic, its effectiveness is generally assessed over several weeks of treatment, rather than immediately after initiation [8]. Monitoring for adverse effects, particularly neuropsychiatric symptoms, is recommended during early treatment, as they may affect patient tolerability and adherence. If mild adverse effects occur, dose or administration time adjustments may be considered to improve tolerability without discontinuing therapy [8].

### 7.5. Role in Multimodal Pain Management

LDN is best considered as part of a broader, multimodal approach to chronic pain management rather than a standalone analgesic [8,12]. The favorable safety and tolerability profile of LDN makes it a reasonable option for consideration in patients who have experienced limited benefit or intolerable adverse effects with conventional analgesic therapies [6,10].

Notably, LDN should not be used concurrently with opioid agonist therapies, as opioid receptor antagonism may reduce opioid analgesic efficacy or precipitate withdrawal symptoms, particularly in opioid-dependent patients [7]. When incorporated into a multimodal pain management strategy, LDN offers a non-opioid agonist approach to symptom management, avoiding direct exposure to the risks associated with long-term opioid agonist therapy. However, further high-quality studies are needed to better define its role in clinical practice [6,8].

### 7.6. Clinical Considerations in Patients Receiving Opioid Therapy

LDN should not be initiated in patients actively receiving daily opioid agonist therapy due to the risk of reduced analgesic efficacy and potential precipitation of withdrawal. In this review, chronic opioid therapy is defined as daily opioid use for ≥3 months. In such patients, opioid tapering should be completed prior to LDN initiation, with tapering individualized according to baseline dose, duration of therapy, and withdrawal risk.

In patients using intermittent short-acting opioids for breakthrough pain, co-administration with LDN may attenuate opioid analgesic effects during periods of receptor blockade. These patients should be counseled regarding the potential for reduced opioid efficacy, and non-opioid rescue strategies should be considered where appropriate.

In perioperative settings, LDN should generally be discontinued in advance of procedures requiring opioid analgesia. Given its relatively short half-life, discontinuation 24–72 h prior to anticipated opioid use may reduce interference with perioperative analgesia; however, data guiding optimal timing remain limited. Re-initiation following acute opioid use should occur only after opioids are discontinued and withdrawal risk has resolved.

## 8. Limitations

The findings summarized in this review should be interpreted in light of important limitations within the current evidence base on low-dose naltrexone (LDN) in chronic pain. Although multiple studies report potential benefits in selected populations, the overall quality and strength of the evidence remain limited.

Much of the clinical literature consists of small randomized trials, pilot studies, retrospective analyses, and case series. Few randomized controlled trials enroll more than 100 participants, and most have follow-up durations of 12 weeks or less, limiting conclusions regarding long-term efficacy and durability of response. Small sample sizes and short follow-up periods contribute to imprecise effect estimates. Many studies are uncontrolled and therefore vulnerable to selection bias, placebo effects, and publication bias.

The included studies vary substantially in design, patient population, dosing strategy, comparator selection, and outcome measures. Few trials report standardized effect sizes or responder analyses. This variability prevents pooling of results across studies and limits cross-condition comparisons. It also precludes conclusions regarding comparative effectiveness relative to established therapies such as serotonin–norepinephrine reuptake inhibitors, tricyclic antidepressants, gabapentinoids, or structured nonpharmacologic interventions. Formal risk-of-bias assessment was not performed, given the narrative design of this review, and study quality was considered descriptively.

Reported efficacy appears to vary by pain phenotype. In fibromyalgia, meta-analytic data suggest a modest benefit compared with placebo. In musculoskeletal conditions such as osteoarthritis, trials have not demonstrated consistent improvement in pain interference. These patterns suggest that future studies should stratify participants by pain phenotype, including nociceptive, neuropathic, and nociplastic categories, and predefine subgroup analyses to clarify whether treatment response differs by mechanism of pain.

There is considerable methodological variability across studies with respect to dosing strategies and treatment duration. Reported LDN doses range from 1 mg to 6 mg daily, and no randomized trial has directly compared fixed-dose versus titrated regimens. Formal dose–response relationships remain insufficiently characterized. Time to clinical response is inconsistently reported, and the durability of benefit beyond several months has not been well defined.

Generalizability is also constrained by study populations that are disproportionately composed of women, particularly in conditions such as fibromyalgia, chronic pelvic pain, and other centralized pain syndromes that are more frequently diagnosed in female patients. Because of this, the applicability of these findings cannot easily be applied to broader chronic pain populations. Furthermore, many studies exclude patients receiving concurrent opioid therapy, which may limit applicability to real-world pain management settings.

While mechanistic hypotheses involving microglial modulation, Toll-like receptor signaling, and central sensitization are biologically plausible, direct human data linking these mechanisms to clinical analgesic effects are scant. As such, conclusions regarding the mechanisms of LDN should be considered inferential.

Collectively, these limitations highlight that current evidence supporting LDN in chronic pain remains preliminary and condition-specific, with inconsistent effect magnitude and limited long-term data. Well-powered randomized trials with standardized outcomes and extended follow-up are required before routine clinical adoption can be recommended.

## 9. Future Directions

Future studies evaluating low-dose naltrexone in chronic pain should prioritize adequately powered, parallel-group randomized controlled trials with predefined primary outcomes and follow-up durations sufficient to assess the durability of response. Trials should stratify participants by pain phenotype and report standardized effect sizes and responder analyses to better identify which patient groups derive clinically meaningful benefit. Mechanistic investigations should incorporate objective measures such as quantitative sensory testing, inflammatory biomarkers, or neuroimaging to determine whether proposed pathways involving microglial activation, Toll-like receptor signaling, and central sensitization are engaged in humans. Such approaches may help identify biological predictors of response. Standardization of dosing regimens and treatment duration is needed. Comparative studies evaluating fixed-dose versus titrated strategies and clearly reporting time to response would improve reproducibility across trials. In addition, longer-term observational studies are required to assess sustained efficacy and long-term safety beyond the short follow-up periods currently available. Finally, randomized studies evaluating LDN as an adjunct to established non-opioid pharmacologic therapies, behavioral interventions, or interventional procedures would help clarify its role within multimodal chronic pain management.

## 10. Conclusions

Low-dose naltrexone represents a repurposed pharmacologic approach that targets mechanisms implicated in chronic pain beyond traditional nociceptive pathways. By influencing neuroimmune signaling, microglial activation, and central sensitization, LDN aligns with current models of chronic pain pathophysiology. Clinical studies suggest potential benefit in selected chronic pain conditions, particularly those characterized by diffuse symptoms and altered central pain processing. However, the current evidence base is limited by small trials and variability within populations and dosing strategies, preventing definitive conclusions on efficacy and optimal use. Within pain practice, low-dose naltrexone may serve as an adjunctive option for carefully selected patients who are not receiving opioid therapy. Its favorable tolerability profile and low cost contribute to clinical interest, but its use should be guided by a cautious interpretation of the available data. Further clinical research is necessary to clarify the therapeutic value of LDN, identify responsive patient populations, and define standardized treatment approaches. Until such data are available, low-dose naltrexone should be considered an emerging therapy within a multimodal framework rather than a standard treatment for chronic pain.

## Figures and Tables

**Table 1:** Comparison of standard-dose naltrexone and low-dose naltrexone.

Feature	Standard-Dose Naltrexone	Low-Dose Naltrexone
Approved indications	Opioid use disorder, alcohol use disorder	None; off-label use
Typical dose range	50 mg orally once daily or 380 mg intramuscular monthly	0.5–4.5 mg orally once daily
Primary therapeutic intent	Sustained opioid receptor antagonism	Modulation of neuroimmune and central pain pathways
Opioid receptor occupancy	Prolonged and near-complete μ-opioid receptor blockade	Transient and partial opioid receptor blockade
Duration of receptor blockade	Continuous	Short-lived, typically several hours
Effect on endogenous opioid signaling	Suppression of endogenous opioid activity	Compensatory upregulation of endogenous opioid signaling after blockade
Proposed central mechanisms	Prevention of exogenous opioid effects	Microglial modulation, reduced neuroinflammation, altered central sensitization
Toll-like receptor (TLR-4) effects	Not a therapeutic target	Proposed antagonism of TLR-4 signaling
Analgesic mechanism	Not intended for analgesia	Indirect modulation of pain processing
Use with opioid agonists	Contraindicated	Contraindicated
Formulation and availability	Commercially manufactured oral and injectable formulations	Requires compounding to achieve sub-therapeutic dosing

**Table 2:** Representative clinical studies evaluating low-dose naltrexone in chronic non-cancer pain conditions. Rows describing meta-analyses and systematic reviews summarize the scope of included primary studies as reported by the authors (number of trials and aggregate sample size where available). These entries contextualize the depth of existing evidence and should not be interpreted as primary interventional trials.

Pain Condition	Study Type	Sample Size	LDN Dose Range	Comparator	Primary Outcomes	Main Findings	Scope of Included Evidence
Fibromyalgia	Randomized, double-blind, placebo-controlled crossover trial [34]	10	4.5 mg daily	Placebo	Self-reported pain severity	Reduced pain severity during the LDN phase compared with placebo	Single randomized, double-blind, placebo-controlled crossover trial (*n* = 10)
Fibromyalgia	Randomized, double-blind, placebo-controlled crossover trial [35]	31	4.5 mg daily	Placebo	Pain, fatigue, stress	Modest improvement in pain and associated symptoms in a subset of participants	Single randomized, double-blind, placebo-controlled crossover trial (*n* = 31)
Fibromyalgia	Systematic review and narrative synthesis [36]	N/A	3–4.5 mg daily	Placebo or usual care	Pain, quality of life	Possible benefit in pain and related symptoms in patient subsets	Included 9 clinical studies (2 randomized placebo-controlled crossover trials and 7 observational or uncontrolled studies), primarily in fibromyalgia; total pooled sample size < 300 across studies
Fibromyalgia	Randomized, double-blind, placebo-controlled parallel-group trial [19]	99	6 mg daily	Placebo	Pain intensity, fibromyalgia impact measures	No significant overall superiority versus placebo; subgroup-level symptom improvement observed	Single randomized, double-blind, placebo-controlled parallel-group trial (*n* = 99)
Fibromyalgia and chronic pain syndromes	Meta-analysis of randomized controlled trials [37]	N/A	1–6 mg daily	Placebo or active comparators	Pain intensity	No significant overall analgesic benefit; modest fibromyalgia-specific effect detected	Meta-analysis of randomized controlled trials Scope of Included Evidence: Included 6 randomized controlled trials (crossover and parallel-group designs) across chronic pain conditions; pooled sample size approximately 250–300 participants; fibromyalgia subgroup analysis performed
Painful diabetic peripheral neuropathy	Randomized, double-blind, active-controlled crossover trial [38]	67	1–4 mg daily	Amitriptyline	Pain scores, adverse effects	Pain relief similar to amitriptyline, with fewer adverse effects	Single randomized, double-blind, active-controlled crossover trial (*n* = 67)
Trigeminal neuropathic pain	Retrospective case series [39]	14	Up to 4.5 mg daily	None	Pain intensity	Reduced subjective pain in refractory cases	Single retrospective case series (*n* = 14)
Complex regional pain syndrome	Systematic review [40]	N/A	3–4.5 mg daily	None	Pain outcomes	CRPS commonly represented in published case reports and series; lack of controlled trials	Included case reports, small case series, and retrospective cohorts; no randomized controlled trials identified; total cumulative sample size < 100 across published reports
Musculoskeletal pain/arthritis	Randomized, double-blind, placebo-controlled crossover trial [41]	23	4.5 mg daily	Placebo	Pain interference	No consistent improvement versus placebo	Single randomized, double-blind, placebo-controlled crossover trial (*n* = 23)
Chronic pelvic pain disorders	Systematic review [42]	N/A	1–4.5 mg daily	Variable	Pain severity, quality of life	Reported reductions in pain and improved quality of life across mixed chronic pain populations that included pelvic pain conditions; pelvic pain–specific efficacy not independently evaluated.	Included mixed chronic pain studies (randomized trials and observational cohorts) in which pelvic pain populations were represented; pelvic pain–specific randomized trials were limited; heterogeneous study designs and small sample sizes
Inflammatory and autoimmune-related pain	Register-based controlled before–after study [43]	10,000	Variable low dose	Pre-LDN baseline	NSAID and opioid use	Reduced dispensing of NSAIDs and opioids following LDN initiation	Single register-based controlled before–after study (*n* = 10,000)
Post-infectious pain (post-COVID)	Observational study [44]	38	1–4.5 mg daily	None	Pain, fatigue	Improvements in pain and fatigue reported	Single observational cohort study (*n* = 38)
Post-COVID condition	Observational cohort study [45]	52	1–4.5 mg daily	None	Symptom burden	Improved pain and functional outcomes reported	Single observational cohort study (*n* = 52)

Notes: All studies administered low-dose naltrexone orally unless otherwise specified. Treatment duration and titration strategies are described in the corresponding text in Section 5; many studies did not employ standardized titration protocols. Where treatment duration or titration strategy was not explicitly reported in the original publication, this is indicated in the text.

## Data Availability

No new data were created or analyzed in this study. Data sharing is not applicable to this article.
